# Behavioural Effects and Naloxone Effectiveness With New Synthetic Opioids

**DOI:** 10.1111/dar.70040

**Published:** 2025-10-15

**Authors:** Suzanne Nielsen, João Pedro Silva, Jermaine D. Jones, Alex Krotulski, Dilkushi Poovendran, Deusdedit Muzangizi, Gilles Forte, Jason White, Sandra D. Comer

**Affiliations:** ^1^ Monash Addiction Research Centre, Eastern Health Clinical School Monash University Melbourne Australia; ^2^ UCIBIO ‐ Applied Molecular Biosciences Unit/i4HB ‐ Associate Laboratory Institute for Health and Bioeconomy, Laboratory of Toxicology, Department of Biological Sciences, Faculty of Pharmacy University of Porto Porto Portugal; ^3^ Division on Substance Use Disorders Columbia University Irving Medical Center & New York State Psychiatric Institute New York USA; ^4^ Center for Forensic Science Research and Education, Fredric Rieders Family Foundation Horsham USA; ^5^ Department of Health Product Policies and Standards World Health Organization Geneva Switzerland; ^6^ Division of Access to Medicines and Health Products World Health Organization Geneva Switzerland; ^7^ Clinical and Health Sciences, University of South Australia Adelaide Australia

**Keywords:** antinociception, naloxone, nitazenes, potency, synthetic opioids

## Abstract

**Introduction:**

The global drug market has seen an emergence of potent synthetic opioids, including benzimidazole‐derived substances known as nitazenes. These compounds have been implicated in fatal and non‐fatal overdoses. This review aims to synthesise current evidence on the pharmacological effects, potency and naloxone responsiveness of new synthetic opioids, with a focus on synthetic opioids evaluated for international control since 2020. It highlights key findings that can inform our understanding of these substances and inform harm reduction strategies.

**Methods:**

A narrative review was conducted using critical reviews from the World Health Organization Expert Committee on Drug Dependence and supplementary literature. Data extraction focused on pharmacological profiles, behavioural effects, potency estimates, and naloxone effectiveness.

**Results:**

Thirteen synthetic opioids were examined, with potencies ranging from similar to morphine to exceeding that of fentanyl. Naloxone demonstrated efficacy in reversing overdose symptoms for most substances, although higher doses or infusions were occasionally required.

**Discussion and Conclusions:**

The variability in nitazene potency and common unintended use underscores the need for widespread education, broad naloxone access and robust drug‐checking initiatives. Key challenges include the detection of nitazenes in polydrug contexts and their presence in substances sold as other drugs, including falsified pharmaceuticals. Emerging evidence suggests that naloxone can reverse acute poisonings with benzimidazole opioids. However, no studies have examined the effectiveness of medications for treating opioid use disorder (e.g., methadone, buprenorphine, naltrexone) in people who are physically dependent on nitazenes. Future research should address this important knowledge gap.

## 1 Introduction

In recent years, a proliferation of synthetic opioids has emerged in the drug market and is being detected in nonfatal and fatal overdoses [1, 2]. This trend began approximately around 2015, with the emergence of fentanyl analogues (e.g., acetylfentanyl) in North America and isolated parts of Europe [1]. Interestingly, fentanyl analogues were not commonly identified in most of Europe (excluding clusters such as those reported in Finland and Estonia), the United Kingdom, or Australia during this initial phase of novel synthetic opioid expansion—their emergence was more closely associated with the United States [3, 4, 5, 6, 7, 8]. As the range of fentanyl analogues (e.g., butyrylfentanyl, furanylfentanyl, methoxyacetylfentanyl) was brought under international control and generic legislation was implemented in some countries (e.g., USA, China), other synthetic opioid substances started to emerge on the global drug market, including the benzimidazolone opioids (e.g., brorphine) and the 2‐benzyl benzimidazole opioids, commonly called ‘nitazenes’ (e.g., isotonitazene, metonitazene) [9].

The nitazenes were initially explored for therapeutic use, but development was discontinued due to adverse effects identified in clinical trials [10, 11]. Limited preclinical and clinical research was conducted prior to abandoning this class of drugs for further exploration for medical use. As a result, most of these substances have not been well characterised. Yet, the increasing detection of nitazenes and other novel synthetic opioids in fatal and nonfatal overdoses underscores the need for a more comprehensive understanding of their effects to inform clinical and policy responses.

In recent years, several in vitro studies have characterised the estimated potencies of these opioids based on binding affinity and efficacy at different opioid receptors [12, 13, 14]. However, with these opioids, in some cases, binding data is not predictive of in vivo potency and efficacy [13]. There have been few attempts to synthesise the behavioural effects of these studies, particularly those that examine agonist effects typically associated with μ‐opioid receptor (MOR) activation. The MOR is primarily responsible for opioid effects including analgesia, as well as sedation and respiratory depression, which are the most clinically relevant adverse effects that contribute to overdose harm [15].

A range of pharmacological approaches exist to characterise the potency and effects of opioids in vivo, with key models focusing on their MOR‐mediated effects. Common experimental study designs in animals are those that examine the antinociceptive (pain‐blocking) effects of opioids, including determinations of their relative antinociceptive potency [16]. The tail‐flick and hot‐plate tests are preclinical models commonly used to evaluate the potential pain‐relieving effects of opioids in rodents. In the tail‐flick test, a rodent's tail is exposed to a focused heat source, and the time it takes for the rodent to flick its tail away indicates its pain threshold. Similarly, in the hot‐plate test, the rodent is placed on a heated surface (with temperatures chosen to measure antinociceptive effects while avoiding permanent damage), and researchers measure the time before the animal exhibits pain responses. These studies can give reasonable estimates of the likely analgesic (and sometimes also sedative effects) of drugs, noting that variations in estimates of potency can occur due to differences in species or in the study model used to test the antinociceptive effects (sometimes called an antinociceptive assay). Various aspects of ‘abuse liability’ can be assessed in rodents by administering controlled doses of opioids and observing behaviours such as self‐administration (to evaluate reinforcing effects). This includes using the two‐lever choice paradigm, where animals trained to self‐administer a known drug are offered a choice between a lever delivering another test substance and a lever delivering a placebo, to assess preference and similarity in reinforcing properties. The drug discrimination test is another model that can be used to assess the similarity of effects between a new drug and one with known abuse potential, like morphine. The animal initially is trained to discriminate the effects of morphine (by responding on one lever) from saline (by responding on a different lever) in order to receive a food pellet. During the training period, food is delivered only if the animal responds on the correct lever. After it reliably discriminates morphine from saline, a test drug can be administered and responding on the morphine versus the saline lever can be measured. Under test conditions, animals receive food pellets regardless of which lever is chosen. Drugs that are morphine‐like will produce almost exclusive responding on the morphine‐associated lever. Additional tests include conditioned place preference (to measure general reward effects), drug discrimination (to assess similarity of pharmacological action and in vivo potency) and withdrawal symptoms (to evaluate physiological dependence) [17].

As substances are identified and considered for national and international control, research is often commissioned to ensure that the pharmacological effects of the substances are adequately characterised to assess whether a substance meets the requirements for placement under national or international control. This research can result in an increased understanding of the pharmacological effects of these emerging substances, which can in turn also inform harm reduction and clinical care in the event of adverse effects or overdose. Given the proliferation of new synthetic opioid substances in the past 5 years, and the need to understand the effects of these potent opioids, this review aimed to provide an overview of the pharmacology of a range of recently identified benzimidazole synthetic opioids, including clarifying relative potencies based on behavioural studies rather than receptor binding studies and identifying evidence gaps. Specifically, this review aimed to summarise in non‐technical language the behavioural pharmacology of new synthetic opioids reviewed with a focus on those considered for international control by the World Health Organization (WHO) since 2020 (since the re‐emergence of nitazenes). This included aiming to document pharmacokinetic profiles, evidence of reversal by naloxone, and any other notable features that might inform clinical and harm reduction responses.

## 2 Methods

A narrative review method was chosen to synthesise the evidence on the pharmacology of synthetic opioids, providing a detailed overview of potency, pharmacokinetics, and naloxone response for clinical and policy contexts. The approach used was a critical narrative review, which involved gathering data from WHO Expert Committee on Drug Dependence (ECDD) Critical Reviews (including information from published Annexes) and supplementary literature to evaluate drug effects [18]. When substances are considered by the WHO for international control, there is a requirement for a review of scientific evidence to occur [19]. The WHO assesses substances for international control by examining scientific evidence relating to the potential for abuse, dependence, and public health risks, alongside any therapeutic value and pharmacological properties of a substance. The WHO also considers the public health impact, patterns of use, pharmacological properties and toxic effects of the substance to determine its overall risk and if it meets the criteria for international control. For all substances under evaluation, the WHO commissions critical review reports to gather and synthesise published and unpublished scientific evidence in addition to collecting data directly through a Member State Questionnaire from all countries in its review process. For this reason, WHO ECDD critical review reports can represent the most complete synthesis of global scientific information relating to emerging substances, and therefore were chosen as the basis for this narrative review. Synthetic benzimidazole opioids that have been considered for international control in the past decade were identified through the ECDD repository [20]. WHO ECDD critical review reports were retrieved and data on pharmacological effects were extracted. Additional searches were conducted in PubMed and Google Scholar in November 2024 using substance names to identify additional papers relating to behavioural pharmacology or overdose management of the synthetic opioids since the publication of the ECDD critical review reports.

Data were extracted by SN using a form that captured the date the substance was initially identified, the date the substance was reviewed, details on behavioural pharmacology studies, any information on relative potency based on behavioural pharmacology studies, evidence of naloxone response and any other clinically relevant details that could inform clinical care in the event of an overdose/poisoning, to understand the context of use or potential harms associated with these substances.

## 3 Results

Thirteen new synthetic opioids were reviewed, including 11 that were reviewed by the ECDD in the past 5 years, in addition to etonitazene and clonitazene, which were controlled at the establishment of the Single Convention on Narcotic Drugs, 1961, and have been included for context (see Table 1).

**TABLE 1:** Summary of key pharmacological characteristics of selected synthetic opioids.

Substance	Year first identified	ECDD review	Potency estimate based on behavioural studies	Potency (behavioural studies)	Onset/duration of action	Naloxone response, and other information on toxicity, use and effects
Etonitazene	1950s	1960	Morphine < fentanyl < etonitazene	Estimated to be 12 times more potent than fentanyl (i.e., hot‐plate test, rats), and 9.5 times greater when measuring catalepsy [13]. For morphine and etonitazene, ED_50_ values were 5.8 and 0.005 mg/kg in the tail‐flick test and 3.1 and 0.003 mg/kg in the hot‐plate test (> 1000× more potent than morphine) [10].	The half‐life (*t*_1/2_) of etonitazene in mice was 1.58 h (95% CI 1.20–2.33) [21]. Maximal antinociceptive effects occurred 15 min after administration and declined faster than morphine [13].	In preclinical studies, antinociception was reduced by the opioid antagonist naloxone [10].
Clonitazene	1950s	1960	Morphine < clonitazene < fentanyl	Antinociceptive potency of clonitazene estimated to be three times greater than morphine [10].	In a clinical trial clonitazene provided analgesia for at least 4 h without typical opioid side effects [10].	No respiratory depression was noted even after repeated doses [10].
Isotonitazene	2019	2020	Fentanyl < isotonitazene	Antinociceptive tests indicate that isotonitazene is three times more potent than fentanyl and many times (estimated to be 1000×) more potent than morphine (hot‐plate test and catalepsy, rat) [13, 22]. Estimated to be 500 times more potent than morphine in a mouse tail‐flick test, and more potent than fentanyl [23].	Onset < 15 min, effects last 120 min 3–10 mcg/kg, return to baseline in 4 h at 30 mcg/kg [24]; *t*_1/2_ = 1.64 h (95% CI 1.34–2.12) in mice [21]; Separately, duration described as being similar to morphine (and therefore heroin), and longer than fentanyl. Sedative effects lasting up to 120 min (morphine started to reduce after 30 min) [22]. Rapidly metabolised (95% in < 60 min) [25], with an estimated *t*_1/2_ of 10 min [25].	Four reports of usual therapeutic doses of naloxone reversing effects of isotonitazene: (1) 4 mg intranasal (IN) + 0.2 mg intravenous (IV) naloxone reversed effects in presence of heroin and fentanyl [26]; (2) fully reversed nonfatal isotonitazene overdose [27]^a^: two overdoses responded to 2 mg intramuscular (IM) and 0.8 mg IV naloxone, though second case required naloxone infusion [28]; (3) naloxone fully reversed isotonitazene overdose [27]^a^, with doses of 0.4–0.8 mg naloxone IV/IM.
Brorphine	2018	2021	Morphine < brorphine < fentanyl	Potency between that of fentanyl and morphine (Sprague–Dawley rats and Swiss‐Webster mice), discriminative stimulus effects of brorphine were used to estimate that brorphine was 9‐times more potent than morphine, and antinociceptive effects in a tail‐flick test showed that the potency of brorphine was between morphine and fentanyl [29].	Brorphine was detected in a serum sample 60 h after emergency department (ED) admission [30] (patient described opioid effects that lasted ‘quite long’). In vitro studies: Maximum effects lasted 75 min and returned to baseline within 120 min [31]. Thermal antinociceptive effects lasted 205 min (had not returned to baseline at 325 min) [32].	Naloxone did not completely attenuate the antinociceptive effects of brorphine in mice, but it fully blocked the respiratory effects of brorphine [32]. A case study indicated a serotonergic syndrome occurred when taken with sertraline [33]. Two cases of brorphine overdose managed in ED indicated reversal with naloxone (doses 0.16 IV and 2 mg IM) [26]. Two further cases of uneventful overdose management with naloxone were reported [34].
Metonitazene	2019	2021	Morphine < metonitazene < fentanyl	In mice, antinociceptive potency is estimated to be approximately 20 times greater than morphine, through other studies estimate it to be 30–100 times the potency of morphine, depending on the route of administration and model used [10, 35]. In morphine‐dependent monkeys, metonitazene was 100 times more potent than morphine in suppressing withdrawal symptoms [22]. In human analgesic trials its analgesic potency was estimated to be 10 times that of morphine [10].	The duration of action of metonitazene is about half that of morphine [22].	A patient died despite a total of 6 mg naloxone (2 mg IM, 4 mg IV); A second patient survived after receiving a total of 10 mg naloxone in 3 doses (4 mg IN for cardiac arrest, 2 mg IN for respiratory depression) [26]; 0.4 mg IV reversed sedative effects, though ongoing respiratory support was required [36]. During preclinical and early clinical trials, effects were reversed by nalorphine (an opioid antagonist considered to be less effective than naloxone) [10, 35, 37]. Naloxone fully reversed nonfatal metonitazene overdose [27]^a^.
Protonitazene	2020	2022	Morphine < protonitazene ≈ fentanyl	Potency measured with ED50 values in an antinociceptive assay (tail‐flick test, rat) comparable to protonitazene (0.035 mg/kg) and fentanyl (0.035 mg/kg) and 140 times that of morphine (4.9 mg/kg) [38].	Rapidly and almost completely metabolised by the human liver (*t*_1/2_ of 9 min) [25].	A case report of a patient who received naloxone (2 mg IM) during an overdose event led to clinical improvement [39]. Naloxone fully reversed nonfatal protonitazene overdose [27]^a^.
Etazene (Etodesnitazene)	2020	2022	Morphine < etazene < fentanyl	Preclinical antinociceptive studies (tail‐flick test, rodent) suggest that etazene is 70× more potent than morphine, but less potent than fentanyl [40, 41].	In a case where naloxone was not administered, a patient required 24 h of mechanical ventilation (suggesting a possible long duration of action), however other substances (flubromazepam, *N‐*ethylpentedrone, and deschloroketamine) were also confirmed to be present [42].	Naloxone fully reversed nonfatal etazene overdose [27]^a^. Preclinical studies demonstrated cardiotoxicity not seen with morphine [43].
Etonitazepyne (*N‐*pyrrolidino etonitazene)	2021	2022	Morphine < fentanyl < etonitazepyne	Etonitazepyne produced dose‐dependent antinociceptive effects (hot‐plate test, Sprague–Dawley rats) [68], with greater potency than morphine and fentanyl (potency in the hot‐plate test was about 10‐ and 2000‐fold higher than fentanyl and morphine) [68]. Antinociceptive effect of etonitazepyne were blocked by naltrexone (warm‐water tail‐flick test) supporting MOR involvement [45].	Dose‐dependent duration of effects of approximately 1 h for lower doses and up to 4 h for the highest doses tested.	A case of acute intoxication reversed by naloxone was reported (no dose described). Identified in falsified oxycodone (in tablets manufactured as falsified Percocet) [46], suggesting its unintentional use may occur among people who purchase pharmaceuticals from unregulated sources.
Butonitazene	2019	2023	Butonitazene < morphine < fentanyl	Antinociceptive potency (tail‐flick, mice) indicated that butonitazene was less potent than morphine and fentanyl [47], but estimated to be more potent than morphine but less potent than fentanyl in drug discrimination tests (two‐lever choice methodology, Sprague–Dawley rats) [48].	Peak antinociceptive effects of butonitazene lasted 90 min and returned to baseline after 180 min [47]. Butonitazene was rapidly and almost completely metabolised by the human liver (*t*_1/2_ = 8 min) [25].	A case report of intoxication reversed by naloxone (2 mg IM) was reported [39].
Etonitazepipne (*N‐*piperidinyl etonitazene)	2021	2024	Morphine < etonitazepipne ≈ fentanyl	Pharmacodynamic evaluation in male Sprague Dawley rats showed that *N‐*piperidinyl etonitazene induces opioid‐like antinociceptive, cataleptic, and thermic effects, its potency in the hot‐plate assay (ED_50_ = 0.0205 mg/kg) being comparable to that of fentanyl (ED_50_ = 0.0209 mg/kg), and > 190 times higher than that of morphine (ED_50_ = 3.940 mg/kg) [44].	The time course of antinociceptive and catalepsy effects at the highest doses (0.1 mg/kg) were up to 4 h, and approximately 1 h for lower doses (0.01–0.03 mg/kg) [44].	Three patients received naloxone with improvement (2 mg IN; 8 mg IN; 0.08 mg IV) [49].
*N‐*desethyl isotonitazene	2022	2024	Morphine < fentanyl < isotonitazene < *N‐*desethyl isotonitazene	Two times more potent than isotonitazene, and seven times more potent than fentanyl in an antinociception test (hot‐plate test, rat), and five times more potent than fentanyl when measuring catalepsy [13]. In a separate antinociception test (tail‐flick test, mice) there was no significant difference in the potency of fentanyl and N‐desethyl isotonitazene [50].	In an animal model the time to the maximal effect was nearly four times longer for *N‐*desethyl isotonitazene (10.5 ± 1 min) than for fentanyl (2.5 ± 0.5 min, *p* < 0.001). The time for recovery from apnoea to baseline respiratory rate was approximately three times longer with *N‐*desethyl isotonitazene (208 ± 38 min) than with fentanyl (67 ± 9 min, *p* = 0.018) [50, 51].	One patient who tested positive only for N‐desethyl isotonitazene presented with coma, miosis, bradypnoea, and hypercapnia and responded to naloxone (1.2 mg IV) pre‐hospital, and infusion (600 μg/h) for 10 h. In a second case of mixed drug toxicity, 0.4–2 mg naloxone was effective in reversing the overdose [52]. Preclinical evidence also supports naloxone reversal of pharmacologic effects [51].
*N‐*pyrrolidino protonitazene (protonitazepyne)	2022	2024	Morphine < fentanyl < *N*‐pyrrolidino protonitazene	*N‐*pyrrolidino protonitazene had considerably greater antinociceptive potency than fentanyl and morphine (tail‐flick test) [53].	Unconfirmed anecdotal reports suggest that its duration of action is longer than fentanyl [53].	2 mg naloxone IM reversed overdoses involving *N*‐pyrrolidino protonitazene (some infusions were required) (webinar communication). A case report noted that naloxone fully reversed *N*‐pyrrolidino protonitazene overdose [27]^a^.
*N‐*pyrrolidino metonitazene (metonitazepyne)	2023	2024	Morphine < *N*‐pyrrolidino metonitazene ≈ fentanyl	*N‐*pyrrolidino metonitazene had similar potency In an antinociceptive assay (tail‐flick test, rodents) as fentanyl [54].	Unconfirmed anecdotal reports suggest that *N*‐pyrrolidino metonitazene has a long duration of action [54].	No descriptions of naloxone use were found.

Abbreviation: ECDD, Expert Committee on Drug Dependence.

^a^ Doses of 400–800 micrograms naloxone IV/IM appear effective from a series of 9 patients involving single nitazene exposures of metonitazene, isotonitazepyne, protonitazene, protonitazepyne, and etodesnitazene [27].

### 3.1 Etonitazene and Clonitazene

**FIGURE 1:**
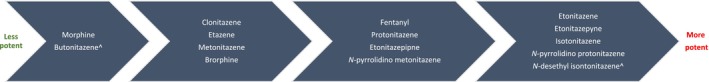
Approximate rank order potency based on behavioural studies (primarily antinociceptive assays; ^potency estimate can vary with assay type).

The first nitazenes considered for international control were etonitazene and clonitazene, considered in 1960 and included in the original list of substances controlled under the 1961 Single Convention on Narcotic Drugs. These substances were part of a group of opioids synthesised by CIBA pharmaceuticals, some of which (including clonitazene) progressed to human clinical trials [40, 55]. In the initial trials of 363 people, clonitazene provided analgesic effects for at least 4 h without exhibiting typical opioid side effects [10]. Despite this, clonitazene was not further developed for therapeutic use [10, 11]. In contrast, etonitazene was estimated to be 10–12 times the potency of fentanyl, depending on the assay, and was estimated to be 1000 times more potent than morphine [10, 13] (see Table 1 and Figure 1). Etonitazene appears to have a relatively fast onset in terms of its analgesic effects, peaking within 15 min of intravenous administration with a duration of action shorter than morphine [13]. In an animal study (Sprague–Dawley rats), etonitazene was found to produce muscle rigidity, potentially similar to the wooden‐chest syndrome produced by fentanyl; however, these same effects were also observed with methadone and morphine (but not codeine) [56].

### 3.2 Isotonitazene

The next nitazene considered for international control was isotonitazene, which was detected in 2019 in North America and Europe and reviewed by the ECDD in 2020. Isotonitazene is now one of the better‐characterised 2‐benzyl benzimidazole opioids. Antinociceptive assays (tail‐flick tests in rodents) demonstrated that isotonitazene was approximately three times more potent than fentanyl, and many times (estimated to be 500–1000 times) more potent than morphine (depending on the species and assay) in tests of antinociception and catalepsy (a rigid immobility where an animal maintains unusual postures) [13, 22, 23]. More than 70 deaths in humans were documented with isotonitazene at the time of its review, with descriptions of being sold as a powder, in falsified Dilaudid (hydromorphone) tablets and as a ready‐made nasal spray product [57]. The descriptions of being sold as a pre‐prepared nasal spray product, and other descriptions of its use in online forums suggest that isotonitazene was intentionally purchased and used, as opposed to this harm relating solely to contamination of other substances with isotonitazene.

### 3.3 Brorphine

Brorphine was first identified in 2018 as a novel psychoactive substance, but was initially synthesised by Janssen pharmaceuticals in 1967 [58], and was reviewed by the ECDD in 2021 [29]. Like most novel synthetic opioids, brorphine is a full MOR agonist but it differs in its chemical structure from nitazenes, being classed as a piperidine benzimidazolone opioid [29]. Brorphine is an example of an opioid where binding data do not predict potency in preclinical behavioural studies. Binding data consistently estimate that it is more potent than fentanyl [59], though behavioural studies show it to be between the potency of fentanyl and morphine [13]. Brorphine appears to have a moderately long duration of action, with analgesic effects lasting more than 3 h and not returning to baseline after 5 h [32]. Online reports suggest that brorphine was commonly used as a drug of choice [29], advertised in unregulated markets as a fentanyl substitute, sold as ‘purple heroin’ (due to the purple colour added to the powder when sold), and has also been detected in counterfeit oxycodone pills [58]. More than 100 fatalities were reported with brorphine in 2020 at the time it was reviewed, with a number of the deaths where brorphine was analytically confirmed describing the cause of death as multiple drug intoxication [29, 60].

### 3.4 Metonitazene

Metonitazene was the next nitazene to emerge. Metonitazene was originally synthesised and explored for therapeutic use in the 1950s [40, 55]. Metonitazene was assessed by the ECDD in the same year as brorphine and appears to have a broadly similar in vivo potency as brorphine, estimated to be between morphine and fentanyl, with a duration of action about half that of morphine [22]. Limited behavioural pharmacology studies on metonitazene exist. It was examined in one clinical trial when it was first explored for therapeutic use, alongside clonitazene and etonitazene [10, 11]. In this study, a 1 mg dose of metonitazene injected subcutaneously or intramuscularly produced analgesia, with side effects of sedation, drowsiness, vertigo, confusion, nausea and vomiting. Respiratory depression with cyanosis was noted to occur in one‐fifth of the patients who received it, leading to the conclusion that further research for clinical use of metonitazene was not warranted [10]. Based on this study, it was estimated to have 10 times the analgesic potency of morphine [10]. More than 20 deaths involving metonitazene were described at the time it was reviewed, including deaths where metonitazene was the only opioid involved and was deemed to be the cause of death [35, 60].

### 3.5 Protonitazene

Protonitazene was identified in 2020 and reviewed by the ECDD in 2022 [61]. In antinociception studies, it appeared to have effects similar to fentanyl and more than 100 times the potency of morphine [38]. It has been detected in drugs sold as ketamine, ‘3‐CP’ (a phenethylamine) and cocaine [61, 62, 63, 64], with the cocaine contaminated with protonitazene identified to be responsible for a multiple fatal overdose event involving four people in Melbourne, Australia [65]. Twenty‐five protonitazene‐related deaths were identified at the time the substance was reviewed, with most deaths reported to involve multiple substances [38].

### 3.6 Etazene (Etodesnitazene)

Etazene shows pharmacological similarities with etonitazene and isotonitazene, having been identified in the illicit drug market in 2020, and reviewed by the ECDD in 2022 [41]. Few preclinical behavioural studies have been conducted with etazene. Studies performed in Swiss Webster mice suggested that etazene was many times more potent than morphine but less potent than fentanyl [40]. In drug discrimination studies performed in rats (two‐lever choice methodology), etazene fully substituted for the discriminative stimulus effects of morphine, demonstrating higher potency than morphine but less potency than fentanyl. Moreover, the administration of naltrexone reduced the morphine‐like discriminative stimulus effects of etazene, further supporting the involvement of MOR on etazene's discriminative stimulus effects [41]. At the time of its review, it was identified in approximately 10 deaths, being reported as the cause of death in at least two of these cases, had been used intentionally (though its use did not appear to be widespread), and was most commonly found in powder and liquid (nasal spray) forms with reported detection in falsified oxycodone tablets [38, 41].

### 3.7 Butonitazene

Butonitazene was first identified in 2019 and reviewed by the ECDD in 2023 [48]. Lab tests using specialised cells engineered to have rat or human opioid receptors showed that butonitazene binds to the MOR with a strength similar to morphine but weaker than fentanyl. However, butonitazene activates this receptor (its ‘agonist effect’) more strongly than either morphine or fentanyl. This suggests that it may produce more potent effects through the MOR, which is the primary receptor involved in opioid pain relief and euphoria. In contrast to this, in behavioural pharmacology studies, butonitazene was shown to produce antinociceptive effects in Swiss‐Webster mice during the warm‐water tail‐flick assay with less potency than morphine or fentanyl. Butonitazene was estimated to be more potent than morphine but less potent than fentanyl in drug discrimination tests (two‐lever choice methodology, performed in Sprague–Dawley rats) [48]. MOR was found to mediate butonitazene's antinociceptive and discriminative stimulus effects, as naltrexone reduced both of these butonitazene‐induced actions [48]. At the time it was reviewed, butonitazene had been identified in one death, along with metonitazene [48]. In this case, the cause of death was attributed to metonitazene overdose [66, 67].

### 3.8 Etonitazepyne

Etonitazepyne (also known as *N*‐pyrrolidino etonitazene) is another nitazene that was originally explored for therapeutic use in the 1950s. It was first detected as an NPS in 2021 and was reviewed by the ECDD in 2022 [46]. Etonitazepyne has also been shown to produce dose‐dependent antinociceptive effects both in Sprague–Dawley rats using the hot‐plate test [68], with higher potency than morphine and fentanyl. For example, its potency in the hot‐plate test was about 10‐ and 2000‐fold higher than fentanyl and morphine, respectively [68]. Etonitazepyne also displayed a profile of cataleptic effects (a rigid immobility) [68], with a dose‐dependent duration of effects (of around 1 h for lower doses and up to 4 h for the highest doses tested). Etonitazepyne has also been shown to fully substitute for the discriminative stimulus effects (two‐lever choice methodology) of morphine in Sprague–Dawley rats, with a higher potency than morphine and fentanyl. Similar to its antinociceptive action, naltrexone blocked the morphine‐like discriminative stimulus effects of etonitazepyne [46].

The intentional use of etonitazepyne has been described, though the extent of use is unclear. At least 29 etonitazepyne‐related fatalities were documented at the time of review, with it being deemed the cause of death in at least three cases [38, 68]. It has been identified in falsified oxycodone (in tablets manufactured as falsified Percocet) [46], suggesting its unintentional use may occur among people who purchase pharmaceuticals from unregulated sources.

### 3.9 *N*‐desethyl isotonitazene

*N‐*desethyl isotonitazene was one of the first nitazene substances that was identified as a novel psychoactive substance that was not initially synthetised in the 1950s [51]. It was first detected in 2022 in the drug market and was reviewed by the ECDD in 2024 [51]. *N‐*desethyl isotonitazene is a metabolite of isotonitazene [67], but it has also been identified in cases where isotonitazene was not present [51, 52]. In behavioural pharmacology studies, *N‐*desethyl isotonitazene produced antinociception in the warm‐water tail‐flick assay (in Swiss‐Webster mice) with higher potency than morphine and fentanyl [51]. In rats, *N‐*desethyl isotonitazene was estimated to be as potent as isotonitazene, and seven times that of fentanyl in an antinociceptive assay using the hot‐plate test, and was estimated to be five times as potent as fentanyl when measuring catalepsy [13]. Drug discrimination studies (using the two‐lever choice procedure) showed that *N‐*desethyl isotonitazene fully substituted for the discriminative stimulus effects of morphine, being more potent than morphine and slightly less potent than fentanyl, with such effects being blocked by naltrexone [51].

At the time of its review, *N‐*desethyl isotonitazene had been confirmed in at least one non‐fatal and nine fatal overdoses, although its contribution to the cause of death was not clear [51]. Moreover, the WHO critical review noted that *N‐*desethyl isotonitazene was identified in 37 post‐mortem cases between 2019 and 2021, although this substance was detected along with isotonitazene, at lower blood concentrations, suggesting that, in such cases, the presence of *N‐*desethyl isotonitazene was most likely due to isotonitazene's metabolism [24]. One feature that may increase the potential danger of this substance beyond its potency is its duration of action, estimated to be approximately four times longer than fentanyl, and taking approximately three times longer to recover from the respiratory depression relative to fentanyl, as determined by comparing the respiratory depressant effects of *N‐*desethyl isotonitazene and fentanyl [50, 51].

*N‐*desethyl isotonitazene has also been identified in counterfeit oxycodone tablets and in samples called ‘dope’ along with other substances (e.g., xylazine, fentanyl, parafluorofentanyl, designer benzodiazepines) [51].

### 3.10 Etonitazepipne (*N*‐Piperidinyl etonitazene)

Etonitazepipne was identified as a novel psychoactive substance in 2021 and reviewed by the ECDD in 2024 [45]. It binds with high affinity to MOR. In rats, etonitazepipne produced potent antinociceptive effects in a hot‐plate assay with potency comparable to fentanyl [44]. It also produced catalepsy and a reduction in temperature.

Etonitazepipne has been identified in several post‐mortem cases [44, 69], although it was only described as the cause of death in three of them and was the sole substance identified in one death [45]. As with etazene, etonitazepipne has also been identified in products sold as oxycodone, suggesting its unintentional use and under‐reported prevalence [70].

### 3.11 *N*‐pyrrolidino protonitazene (Protonitazepyne)

*N‐*pyrrolidino protonitazene was identified in the drug market in 2022 and reviewed by the ECDD in 2024 [53]. In an antinociceptive assay, *N‐*pyrrolidino protonitazene had considerably greater potency than fentanyl and morphine, with unconfirmed anecdotal reports suggesting it may have a longer duration of action than fentanyl [53]. *N‐*pyrrolidino protonitazene was identified in 39 deaths at the time of its review in 2024, with most evidence suggesting its use was unintentional; for example, individuals report using drugs sold as heroin or fentanyl. *N‐*pyrrolidino protonitazene was responsible for one of the largest overdose clusters documented, confirmed in 57 cases in Dublin and 20 cases in Cork, Ireland, between November and December 2023, respectively [71].

### 3.12 *N*‐pyrrolidino metonitazene (Metonitazepyne)

*N‐*pyrrolidino metonitazene was identified in the drug market in 2023, and reviewed by the ECDD in 2024 [54]. Due largely to its relatively recent detection, there is limited information on the effects or use of *N‐*pyrrolidino metonitazene. In an antinociceptive assay (tail flick) *N‐*pyrrolidino metonitazene appeared to have similar potency to fentanyl, with unconfirmed anecdotal reports suggesting it may have a longer duration of action than fentanyl [54]. At the time of its review, *N‐*pyrrolidino metonitazene had been detected in 15 deaths, though other opioids were detected in almost all of these deaths. Information from online forums suggests that *N‐*pyrrolidino metonitazene may be used intentionally, with no reports of it being sold as other substances, though its use does not appear to be widespread.

### 3.13 Relative Potency

In Figure 1, we represent the approximate relative potency for these substances in order from least to most potent, noting that for some substances, depending on the assay used, there is a variation in their order of potency based on behavioural studies, with butonitazene being less potent, similar to morphine, and five substances (etonitazene, isotonitazene, *N‐*pyrrolidino protonitazene, etonitazepyne and *N‐*desethyl isotonitazene) estimated to be more potent than fentanyl.

### 3.14 Naloxone Response

Evidence of the use of naloxone extracted from identified papers suggests that for almost all of the synthetic opioids described in this review, naloxone was effective in usual therapeutic dose ranges (see Table 1). Interpretation of this data, however, is complicated by the fact that many poisonings involved multiple opioids, benzodiazepines and other substances.

For clonitazene, no reports of naloxone use were identified, but reports of its use in initial clinical trials suggested that no respiratory depression was observed with single or repeated doses [10]. For even the most potent nitazenes, there was evidence of naloxone effectiveness in reversal of overdose in humans [52] alongside preclinical evidence that supported naloxone antagonism of opioid agonist effects [51]. In some clinical cases, naloxone infusions were required, and there were some documented cases of fatalities despite naloxone administration.

## 4 Discussion

This narrative review collated available information on the pharmacological effects of 11 synthetic opioids that have been recently considered for international control, including evidence of the effectiveness of naloxone in reversing their effects. Key findings include the wide range of potencies observed in behavioural studies that might most closely approximate the potency of these synthetic opioids in humans, from being less than morphine (depending on the model used) to many times more potent than fentanyl. These potencies can vary greatly by species, and on the assay used to assess potency. For example, depending on the antinociceptive assay, *N‐*desethyl isotonitazene had either comparable potency to morphine or 10‐fold higher potency [13, 50]. For this reason, relatively broad groupings of potency are presented, rather than specific rank‐order potency for each opioid.

It is also important to note that although antinociceptive potency is usually correlated to respiratory depressant effects, there are a range of complex mechanisms that may influence the respiratory depressant effects of opioids with extensive research considering and questioning the importance of β‐arrestin mediated versus G protein pathway‐mediated effects, which are beyond the scope of this review (see [72, 73] for more detailed discussions). The majority of studies identified in this review examined antinociceptive effects without directly measuring respiratory effects, and these were mostly studies conducted in rodents, with clinical studies in humans limited to reports from studies in the 1950s.

Further, although nitazenes are often described as significantly more potent than other opioids, potency alone does not equate to greater harm, and as described in this review, the potency of nitazenes in some cases is similar to that of opioids like morphine which are routinely used in therapeutic settings. Some care is needed to avoid alarmist narratives in the media, which can drive criminal and political responses [74], and to also acknowledge that risk emerges from the unpredictable and unregulated nature of the drug supply, where variability in concentration, dilution and adulteration increases the risks associated with the often‐unexpected presence of these substances. The context of use also varied greatly with some opioids being used intentionally, for example, with isotonitazene where an illicitly manufactured nasal spray with pre‐measured doses was being sold via the internet. Other nitazenes, such as protonitazene have been mis‐sold as, or identified in other substances including 3C‐P and cocaine, and ketamine. *N‐*desethyl isotonitazene, etazene or etonitazepyne, have also been found in falsified oxycodone and benzodiazepine tablets, and nitazenes have been detected in liquids intended for vaping [75]. This has important implications for the availability and promotion of drug checking as a harm reduction measure, and for naloxone provision and education in much broader treatment or harm reduction settings where people use any illicit drugs, including younger populations who may use vaping products, rather than limiting the focus of these programs to those people who may intentionally use opioids. Similarly, because people with chronic pain and other populations who use pharmaceutical opioids report greater difficulty accessing opioids through medical systems, the risk that people may seek pharmaceutical opioids online or through unregulated sources is likely to drive demand for falsified pharmaceutical opioids, which may in turn drive greater harm with these products.

Due to the high potency and structural variability of nitazenes, their detection presents significant challenges. The reliability of many portable drug‐checking technologies is limited to substances that are present in very low concentrations [76]. Although nitazene‐specific test strips are available, they do not detect all analogues within the class, and false positives have proven to be a concern with fentanyl test strips [77]. Consequently, access to fixed‐site drug‐checking services equipped with advanced analytical instrumentation, such as gas or liquid chromatography coupled with mass spectrometry, is essential for accurate detection and harm reduction.

Despite the wide‐ranging potencies of these opioids, emerging evidence of the ability of naloxone to reverse acute poisoning in humans was identified for almost all of the opioids examined, with naloxone doses (when reported) typically representing doses in the usual therapeutic range, suggesting that naloxone remains a key overdose reversal agent and harm reduction tool in drug markets where nitazenes are emerging. This is consistent with a recent case series of 32 nitazene poisonings in Australia that also found standard naloxone doses to be effective [78]. Our understanding of naloxone for reversing effects of nitazenes is likely to continue to evolve, and will be important to inform harm reduction work. An ongoing study collating a case series of patients positive for novel synthetic opioids [26] can contribute to a better understanding of naloxone effects in real‐world settings as further cases are identified. Similarly, work conducted by NSW Health in Australia as part of the Prescription, Recreational and Illicit Substance Evaluation (PRISE) Program [79] was able to demonstrate that standard doses of naloxone appear effective based on analysis of a series of nine cases involving single nitazenes. There are challenges in interpreting *why* naloxone was not able to reverse opioid effects in some instances. In these cases, it may be unknown how long a person was unconscious or not breathing before naloxone was first administered, or before medical help arrived, and interpretation is further complicated by the common detection of multiple opioids in most cases. It is possible in some cases that due to the time passed or the dose of opioids taken, naloxone reversal of opioid symptoms may not have been possible irrespective of the dose administered, and the administration of high naloxone doses (resulting in over antagonism and related harm [80]) should be balanced with the risk of providing a lower initial dose and then requiring additional doses. Similarly, nalmefene has been proposed as an alternative for higher potency opioids, but the long half‐life and risk of precipitating severe withdrawal [81] may limit its utility, particularly given the emerging evidence described here supporting that naloxone can be used effectively with nitazenes, and that naloxone and nalmefene are equipotent in reversing fentanyl overdose [82]. The role of oxygen support is also an important consideration for overdoses on high potency opioids, with opioids like fentanyl being more potent than morphine in depressing brain oxygen concentrations [83]. Hypoxia is the primary cause of death in opioid overdose, and while naloxone remains the standard of care for reversal, as noted in our findings, there may be cases where it is less effective or requires repeated dosing in the context of ultra‐potent opioids. As such, ventilation with oxygen will remain a critical component of overdose response, particularly where naloxone is unavailable or is not sufficient to maintain respiration [84]. For example, standard basic life support guidelines in Australia (Australian and New Zealand Committee on Resuscitation) advise administration of supplementary oxygen when available and when trained to do so during opioid overdose response [85]. While oxygen does not reverse opioid receptor‐mediated effects, it preserves tissue oxygenation and supports vital functions when naloxone may be delayed, unavailable or less effective and, as such, should be central in discussions about responding to nitazene‐related harm, including considering adaptations of protocols for these contexts.

Similarly, given their long half‐life, unclear pharmacokinetics and common adulteration with novel benzodiazepines, treatment approaches for nitazene dependence are likely to require adaptation to address current gaps in knowledge. Evidence suggests that opioid agonist treatment is effective against fentanyl and important in reducing mortality [86, 87], but it may be less effective in treating people with opioid use disorder who use fentanyl compared to heroin, and induction onto it may be more complicated. The same may be true of nitazenes, especially those nitazenes that are more potent. The impact on areas such as pain management for people dependent on nitazenes also remains unknown. Further, a focus on reducing barriers to accessing evidence‐based treatments for opioid dependence, including cost, stigma and geographical barriers, will remain critical given the increased risks of unregulated drug use and overdose for people who are out of treatment.

Of note, all of the opioids reviewed here were recommended for international control. The extent to which national and international drug control measures influence the availability of new synthetic opioids, such as nitazenes, remains unclear. It is yet to be determined whether these controls will limit the synthesis of new nitazene analogues or alter the availability of already controlled opioids; however, recent trends suggest that novel nitazenes may continue to emerge [88]. The international control (including country‐level generic legislation) of fentanyl and its analogues appeared to precede the emergence of nitazenes, suggesting that perhaps those synthesising these unregulated opioids are developing newer opioids to avoid criminal penalties. The recent decision by Chinese authorities to schedule the entire nitazene class may have important implications for global supply and could influence future trends in the appearance of these substances [89].

In conclusion, there has been a rapid emergence of the benzimidazole synthetic opioids, a group of drugs with wide variation in potency and effects. The common identification of these substances in drugs sold as stimulants, or in falsified pharmaceutical opioids and benzodiazepines means that education of the general population across broad age ranges and settings, about the risks of these substances should be a priority. Although it is reassuring that naloxone appears to be effective in reversing overdoses involving this group of opioids, the effectiveness of medications for treating opioid use disorder (buprenorphine, methadone or naltrexone) in people who use nitazenes has not been examined in either preclinical or clinical studies, highlighting this as an important area for ongoing clinical research.

## Author Contributions

Suzanne Nielsen, Jason White, and Sandra D. Comer conceptualised the paper. Suzanne Nielsen led the writing of the manuscript, conducted the literature searches and data extraction, and oversaw the overall development of the paper. João Pedro Silva contributed drafting of manuscript sections and interpretatoin of the data. Jermaine D. Jones, Alex Krotulski, Dilkushi Poovendran, Deusdedit Muzangizi, and Gilles Forte contributed to interpretation of the evidence, provided critical revisions, and approved the final version of the manuscript. All authors reviewed the manuscript for intellectual content and approved the submitted version.

## Conflicts of Interest

S.N. has served as a consultant/temporary advisor to the WHO. S.N, J.P.S., A.K., J.D.J. and S.D.C. have written critical review reports commissioned by the WHO's Expert Committee on Drug Dependence. J.W. has served as a consultant to WHO. In the past 3 years, S.D.C. has received grant support through the National Institute on Drug Abuse for studies in collaboration with BioXcel Therapeutics, GoMedical, Intracellular Therapies, and Lyndra Therapeutics. She also received direct research support from BioXcel Therapeutics and Janssen, as well as consulting income from Alkermes, Lykos Therapeutics, Syneos Health, and Zevra Therapeutics. In the past 3 years, J.D.J. has received compensation from the American Psychological Association, the AIDS Education and Training Center, and BioXcel Therapeutics.

## Data Availability

Data sharing not applicable to this article as no datasets were generated or analysed during the current study.
